# NiFe-based Prussian blue analogue nanopolygons hybridized with functionalized glyoxal polymer as a voltammetric platform for the determination of amisulpride in biological samples

**DOI:** 10.1007/s00216-023-04559-0

**Published:** 2023-02-21

**Authors:** Marwa R. El-Zahry, Marwa F. B. Ali

**Affiliations:** 1 Pharmaceutical Analytical Chemistry Department, Faculty of Pharmacy, Assiut University, Assiut, 71526 Egypt; 2 Pharmaceutical Chemistry Department, Faculty of Pharmacy, Badr University in Assiut, Assiut, 2014101 Egypt

**Keywords:** Amisulpride, Bimetallic (NiFe) Prussian blue analogue nanopolygons, Glyoxal polymer nanocomposites, *In situ* electro-polymerization, Square wave voltammetry

## Abstract

A novel voltammetric platform based on pencil graphite electrode (PGE) modification has been proposed, containing bimetallic (NiFe) Prussian blue analogue nanopolygons decorated with electro-polymerized glyoxal polymer nanocomposites (p-DPG NCs@NiFe PBA Ns/PGE). Cyclic voltammetry (CV), electrochemical impedance spectroscopy (EIS), and square wave voltammetry (SWV) were utilized to investigate the electrochemical performance of the proposed sensor. The analytical response of p-DPG NCs@NiFe PBA Ns/PGE was evaluated through the quantity of amisulpride (AMS), one of the most common antipsychotic drugs. Under the optimized experimental and instrumental conditions, the method showed linearity over the range from 0.5 to 15 × 10^−8^ mol L^−1^ with a good correlation coefficient (*R* = 0.9995) and a low detection limit (LOD) reached, 1.5 nmol L^−1^, with excellent relative standard deviation for human plasma and urine samples. The interference effect of some potentially interfering substances was negligible, and the sensing platform demonstrated an outstanding reproducibility, stability, and reusability. As a first trial, the proposed electrode aimed to shed light on the AMS oxidation mechanism, where the oxidation mechanism was monitored and elucidated using the FTIR technique. It was also found that the prepared p-DPG NCs@NiFe PBA Ns/PGE platform had promising applications for the simultaneous determination of AMS in the presence of some co-administered COVID-19 drugs, which could be attributed to the large active surface area, and high conductivity of bimetallic nanopolygons.

**Graphical Abstract:**

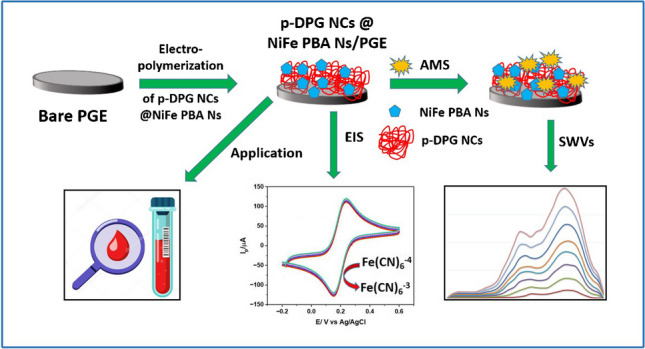

**Supplementary Information:**

The online version contains supplementary material available at 10.1007/s00216-023-04559-0.

## Introduction

Many medications are being authorized for the treatment of COVID-19 that are based either on a compassionated use or as part of randomized controlled trials. Antipsychotics are usually prescribed for the pharmacological treatment of confusion and behavioral disturbances accompanied with hospitalization of COVID-19 patients, especially elderly cases [1]. It has been reported that COVID-19 medications may have serious adverse effects and potential drug interactions with some antipsychotic agents, such as QTc and/or PR prolongation and neutropenia [2]. One of the most common antipsychotics that may be used concurrently with drugs, to treat COVID-19 symptoms, is amisulpride (AMS), a well-established antipsychotic. From this point of view, a highly sensitive and reproducible analytical method is mandatory for close monitoring and accurate controlling of AMS level in biological samples [3].

AMS is widely used for effectively treating schizophrenia. It has unique typical antipsychotic profile, and favorable side effect profile for selected patients. AMS is a second-generation antipsychotic drug that is classified as a substituted benzamide. On low-dose application, it binds with D2/D3 presynaptic autoreceptors, increasing dopamine transmission in the prefrontal cortex, improving the negative symptoms. While at higher doses, antagonism of postsynaptic dopamine receptors occurred allowing their effects in improving positive symptoms [4].

Besides the potential adverse interactions of AMS with the COVID-19 medications, AMS is generally well tolerated and its neurological tolerability profile is superior to that of other conventional antipsychotics. AMS overdose may induce seizures, QT prolongation, and torsades de pointes; its long-term use leads to several side effects such as glucose metabolism alteration, coronary heart diseases, and osteoporosis [5].

Literature has contained several analytical methods for determination of AMS in biological samples, most notably chromatographic [6–9], spectrophotometric [10–12], and spectrofluorometric [13, 14]. Also, a few works based on electrochemical methods have been reported for AMS using ion-selective electrode [15] or unmodified glassy carbon electrode [16], and modified electrode with fsDNA [17], or ruthenium nanoparticles [18]. The electrochemical methods received the most attention owing to their excellent sensitivity, efficiency, accuracy, and cost-effectiveness. Additionally, they are facile to use, compatible with miniaturization, and portable with instrumentation and exhibit the ability of improving the sensitivity and selectivity of the quantitative analysis through using different modified surface electrodes [19].

Based on promising applications in the design of multifunctional substances, Prussian blue analogues (PBAs), one of the metal–organic frameworks’ (FWMs) members, are still being investigated because of their tunable, open structures allowing insertion of species. Due to their superior characteristics such as low cost, straightforward synthesis, and stability, PBAs have been used in various applications, such as environmental purification [20] and drug delivery [21].

Compared with single central metal PBAs, bimetallic PBAs exhibited better behavior owing to the synergistic effect of both metals’ counterparts. The open framework structure of bimetallic PBAs offers several advantages, including greater durability, faster charge transfer, and significant magnetic properties. Bulk synthesis of PBAs has been performed by spontaneous precipitation methods. The reason for limiting their application is their poor conductivity in both acidic and alkaline media. In order to overcome this limitation, we tried to hybridize the polymer nanocomposites with PBA nanomaterials to enhance the electrochemical properties of the sensing platform.

Due to their distinguished electrical, optical, and chemical characteristics, conducting polymers, having extended π-conjugated systems, have been broadly utilized in the synthesis and preparation of electrochemical sensors owing to their high conductivity, ease of polymerization, and redox property. In order to expand the electrochemical application of the conducting polymer, their nanocomposites with inorganic nanoparticles were incorporated [22, 23]. This incorporation does not only improve polymer characteristics, but also provides a new performance due to the synergistic effect of its components which cannot be achieved in their single case.

Electrochemical polymerization is based on the deposition of a polymer onto the surface of a solid electrode material or nanomaterial. Electrochemical polymerization of electroactive monomers occurs in the presence of inorganic NPs, clusters, or metal–organic frameworks (MOFs) [24]. Pencil graphite electrode (PGE) gave good results owing to its remarkable features such as high electrochemical reactivity, low background current, good mechanical rigidity, ease in modification, low cost and commercial availability [25–29].

Inspired by the previous literature, a novel, highly sensitive, and selective voltammetric sensor was adopted in this study. This sensor is based on hybridization of bimetallic (Ni–Fe) Prussian blue analogue nanopolygons with polymer DPG nanocomposites (p-DPG NCs@NiFe PBA Ns/PGE). The synergistic effect of PBA nanopolygons with DPG nanocomposites has remarkably improved the electrochemical activity towards AMS electro-oxidation, allowing us to reach a LOD up to 1.5 nmol L^−1^. The formation of p-DPG NCs@NiFe PBA Ns was confirmed using different analytical techniques, including PXRD, SEM, TEM, CV, EIS, and SWV methods. Additionally, the electrochemical aspects of the modified platform were further investigated in monitoring the electro-oxidation of AMS in nanoscale level in different biological samples. The selectivity of the sensing platform was investigated via monitoring SWVs of AMS in the presence of some co-administered COVID-19 medications.

## Experimental

### Materials and reagents

Amisulpride (AMS, purity 98%) and 2,2′-dipyridyl glyoxal (DPG) were provided by Sigma-Aldrich, Cairo, Egypt, and were used as received. Nickel nitrate, sodium citrate, and potassium hexacyanoferrate were obtained from Sigma-Aldrich. The voltammetric study was carried out in the pH range from 5.5 to 9.0 using Britton-Robinson (BR) buffer. Double-distilled water was used for the preparation of different buffer solutions, standard solutions, and electrolytes. All chemicals were of analytical reagent grade and were used as received.

### Instrumentation

All the electrochemical measurements were performed using a Princeton VersaSTAT MC (VersaSTAT 3, Model RE-1, Princeton Applied Research, AMETEK, USA) controlled by 394 software in conjunction with a PAR Model polarographic analyzer equipped with a three-electrode cell incorporating bare or modified pencil graphite electrode PGE as a working electrode, Ag/AgCl (3.0 M KCl) as a reference electrode, and a platinum wire as an auxiliary electrode. Z-view software (Z-view version 3.5d) was utilized for electrochemical impedance spectroscopy calculations.

Powder X-ray diffraction (PXRD) (PW 2103 Philips diffractometer (*λ* = 1.5418 Å)) operated at 35 kV and 20 mA stimulated by Cu Kα radiation (Ni‐filtered) over the range 10–80° (2*θ*).

A Nicolet 6700 FTIR Advanced Gold Spectrometer, supported with OMNIC 8 software (Thermo Electron Scientific Instruments Corp., Madison, WI, USA), was used for data processing. Both intact and oxidized AMS samples to be analyzed were mixed with potassium bromide (KBr), without any pre-treatment stages. The oxidized AMS was collected from the surface of the modified electrode by scratching the pencil electrodes after each electro-oxidation process. All experiments were performed in triplicate.

A scanning electron microscope (SEM), JEOL JSM-5400 LV instrument (Oxford, USA), and a transmission electron microscope (TEM), FEI TECNAI F20 microscope, were used for monitoring the surface modification of the modified electrode.

### Fabrication of p-DPG NCs@NiFe PBA Ns/PGE

By using a co-precipitation method, aqueous precursors containing 40 mM of Ni(NO_3_)_2_ and 20 mM of K_3_Fe(CN)_6_ were prepared by dissolving the solid precursors in double-distilled water under magnetic stirring forming solution **A**. Sodium citrate, 0.365 g, was dissolved in double-distilled water (75 mL) to form solution **B**. Then, solution **B** was added to solution **A**, obtaining a green homogeneous mixture. The obtained mixture was further stirred for 15 min. at 70 °C (Scheme S1). The solid precipitate (Ni-Fe PBA Ns) was collected, washed several times with ethanol, and dried at 60 °C for subsequent structural characterization and applications.

The electrochemical method was applied for preparation of polymer nanocomposites using a polarographic analyzer which was equipped with a three-electrode cell. All the experiments were carried out in a one-compartment cell at room temperature. The electrochemical polymerization of DPG and deposition of NiFe PBA Ns on the PGE electrode were done potentiostatically by applying a fixed potential of + 2.5 V for 120 s in ethanol solution (10 mL) containing 20 mg of DPG and 40 mg of NiFe PBA Ns. After polymerization, the polymer-coated PGE substrate was washed with ethanol to remove any residue. The electrode was denoted with p-DPG NCs@NiFe PBA Ns/PGE.

### General analytical procedure

For determination of AMS, 6-mL of BR buffer (pH 8.0) containing a suitable amount of AMS was added to the electrochemical cell. The voltammograms were recorded using p-DPG NCs@NiFe PBA Ns modified electrode. The developed SWV plots were monitored using a potential range of 0.0 to +1.5 V with different experimental parameters for optimization procedures.

EIS measurements for bare PGE and p-DPG NCs@NiFe PBA Ns electrodes were monitored in the frequency range 1 Hz to 10 kHz using Princeton Versa STAT MC. The impedance spectra were recorded applying 10 mV amplitude as a formal potential of the redox pair [Fe(CN)_6_]^−3^/^−4^ at a concentration of 0.2 mmol L^−1^ in 0.1 mol L^−1^ KCl solution. In order to obtain the key figures of merit, EIS results were fitted to Randles equivalent circuit and obtained as Nyquist plots using Z-view software.

### Spiked-sample analysis

Human urine and plasma samples from the blood bank have been granted by Assiut University Hospitals (Assiut, Egypt). The pooled plasma samples have been collected from healthy volunteers and stored under -20° C until use. For the determination of AMS in spiked human plasma, each 0.5 mL of human plasma was transferred to a 2.0-mL Eppendorf tube, spiked with different concentrations of AMS, followed by addition of 1.0 mL of acetonitrile to remove the interfering proteins. After 5-min vortex, the obtained solutions were centrifuged at 4000 rpm for 20 min. The supernatant was collected and used for further analysis. A suitable volume was transferred into the electrochemical cell containing the selected supporting electrolyte, and the analytical procedure was conducted as previously mentioned in the general procedure section.

Human urine samples were prepared by diluting samples five times with double-distilled water followed by addition of 1 mL of AMS standard solutions with different concentrations. The quantitative estimation of AMS was conducted following the previously described procedure.

## Results and discussion

### Characterization of the p-DPG NCs@NiFe PBA Ns

**Fig. 1:**
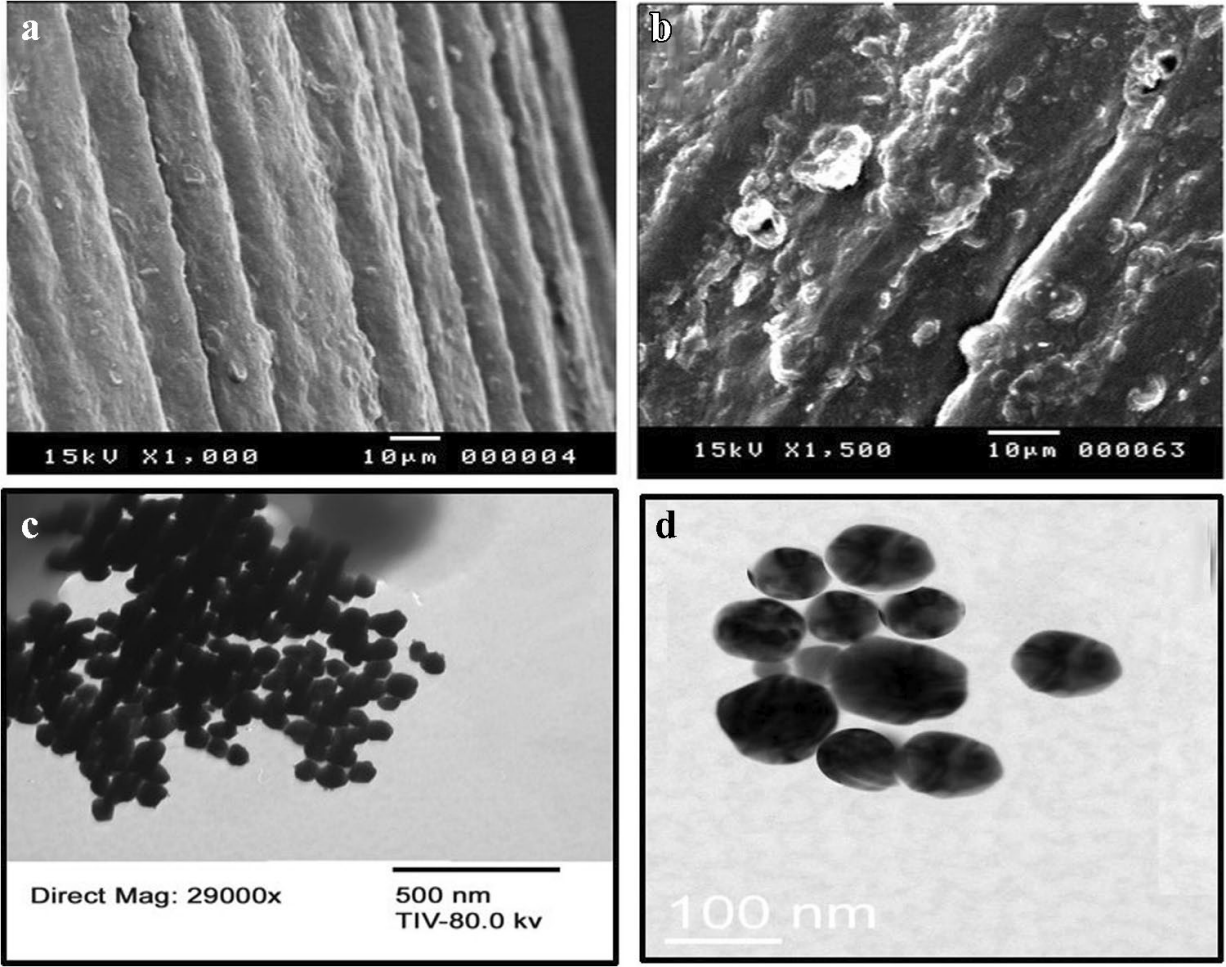
SEM images of (**a**) bare PGE and (**b**) p-DPG NCs@NiFe PBA Ns, (**c**) TEM image of NiFe PBA Ns, and (**d**) magnified TEM image of NiFe PBA Ns

SEM technique was applied to illustrate the morphology of bare PGE, NiFe PBA Ns, and p-DPG NCs@NiFe PBA Ns. As shown in Fig. 1a, the surface of the bare electrode is shown to be smooth, free from any projections. After incorporation of p-DPG NCs conjugated with metallic NiFe PBA nanopolygons, the surface was covered with bigger and uneven nanostructures, because of the formation of the poly-DPG membrane (Fig. 1b). In order to confirm the synthesis of NiFe PBA nanopolygons, TEM images with two magnifications were recorded (Fig. 1c, d). The results confirm that the synthesis of nanopolygons with an average particle size of 70 nm was successfully applied leading to an increase in the active surface area and an increase in the adsorption of the targeted species at the modified electrode.

The structural information of the as-prepared nanocomposites was investigated using powder X-ray diffractometry (PXRD). The XRD patterns of both NiFe PBA Ns and p-DPG NCs@NiFe PBA Ns are presented in Fig. S1 showing the peaks at 2*θ* = 44.4°, 52.03°, and 76.84° which can be respectively indicated by (111), (200), and (220) planes of NiFe PBA Ns in a hexagonal crystal. After the incorporation of DPG NCs, diffraction peaks of the polymer appeared. The PXRD pattern p-DPG NCs@NiFe PBA Ns shows the peaks at 2*θ* = 20.86° and 25.72° that are characteristic diffraction peaks of the DPG NCs [30].

**Fig. 2:**
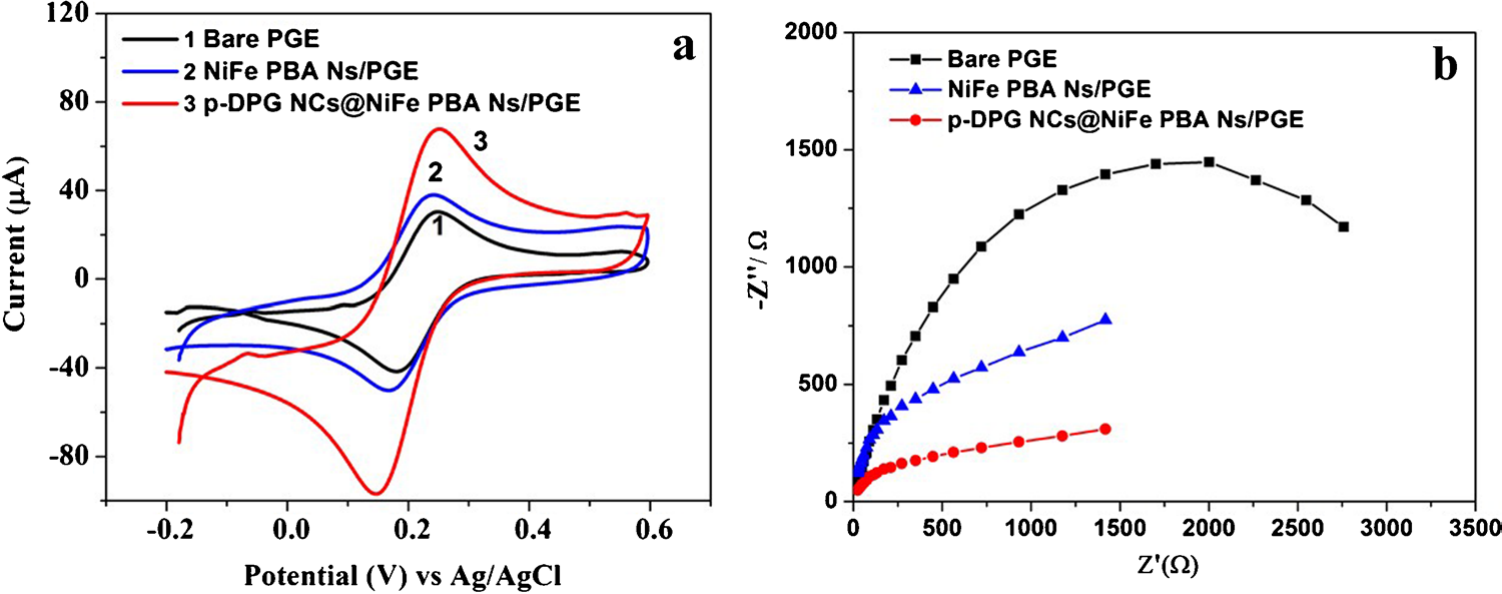
**a** CV curves and **b** Nyquist plots of 1 mmol L^−1^ Fe^2+^/Fe^3+^ mixture in 0.5 mol L^−1^ KCl monitored on bare PGE, NiFe PBA Ns/PGE, and p-DPG NCs@NiFe PBA Ns/PGE. Working conditions: scan rate 0.1 Vs^−1^ and potential range − 0.2 to + 0.6 V

The electrochemical impedance spectroscopy (EIS) and cyclic voltammetry (CV) were used to describe the surface modification and the interfacial properties of PGE with modified nanocomposites, namely, bare PGE, NiFe PBA Ns, and p-DPG NCs@NiFe PBA Ns. Figure 2a represents cyclic voltammograms of 1.0 mmol L^−1^ Fe^2+^/Fe^3+^ redox probe in 0.5 mol L^-1^ KCl. Considering the bare PGE, a well-defined voltammogram with symmetric anodic and cathodic peaks and the peak separation (Δ*E* = 70 mV). The deposition of NiFe PBA Ns on the PGE resulted in a slight increase in the current density with a growth of Δ*E* to 80 mV. After modification of p-DPG NCs@NiFe PBA Ns, a distinct improvement of the electrochemical effect of the modified electrode was observed in terms of higher peak current intensities and a great shift in Δ*E* to 100 mV between the anodic and cathodic peaks of Fe^2+^/Fe^3+^ system. This enhancement may be attributed to the increase in active surface area of the modified surface electrode. These results were further confirmed with EIS measurements.

The interfacial properties of the modified electrodes were investigated using the EIS technique. Figure 2b shows the Nyquist plots of bare PGE, NiFe PBA Ns, and p-DPG NCs@NiFe PBA Ns. The EIS experiments were carried out using 1.0 mol L^-1^ Fe^2+^/Fe^3+^ mixture in 0.5 mol L^-1^ KCl. It was performed at a potential amplitude of 10 mV over a frequency range of 1.0 Hz and 10 kHz. According to the obtained EIS spectra, the characteristic parameters such as the charge transfer resistance (*R*_ct_), the diameter of the semi-depressed circle corresponding to the charge transfer process across the PGE electrode, and the generalized Warburg impedance (Ws) for finite diffusion of the reactants were calculated using Zview software (Zview version 3.5d). The recorded EIS spectra show that the modification of PGE with NiFe PBA Ns results in a slight decrease in *R*_ct,_but to a lesser extent than in the case of p-DPG NCs@NiFe PBA Ns, which proves that the introduction of the conducting polymer DPG NCs to NiFe PBA Ns facilitates the electron transfer and decreases the charge transfer resistance (the lowest *R*_ct_ value) allowing rich electron conductivity to the modified electrode.

The Nyquist plots of the modified electrodes show a linear shape indicating a diffusion behavior compared with the bare PGE electrode which shows a clear circular curve. The enhancement of the electro-activity of the modified electrode may be due to the speed in the charge transfer and the increase in the surface area. In order to confirm this proposal, the active surface area of the studied electrode has been calculated according to the slope of the anodic peak current (*I*_p_) using Randles-Ševćik equation [31]:

$${I}_{\mathrm{p}}=\left(2.69\times {10}^{5}\right){n}^{3/2}{A}_{\mathrm{eff}}{{D}_{R}}^{1/2}{C}_{0}{\upsilon }^{1/2}$$

where *n* is the number of electron transfer, *A*_eff_ is the surface area of the electrode (cm^2^), *D*_R_ is the diffusion coefficient (cm^2^ s^−1^), *υ* is the scan rate (V s^−1^), and *C*_0_ is the concentration of the Fe^2+^/Fe^3+^ mixture (mol cm^−3^). According to the Randles-Ševćik equation, the active surface area of bare PGE and p-DPG NCs@NiFe PBA Ns electrodes has been calculated to be 43.0 and 153.2 mm^2^, respectively. The results indicated the enhancement effect of the hydride nanocomposites with the Prussian blue analogue nanopolygons, allowing the increase in the active sites and improving the adsorption of AMS on the fabricated electrode.

### The electrochemical behavior of AMS towards the p-DPG NCs@NiFe PBA Ns modified electrode

**Fig. 3:**
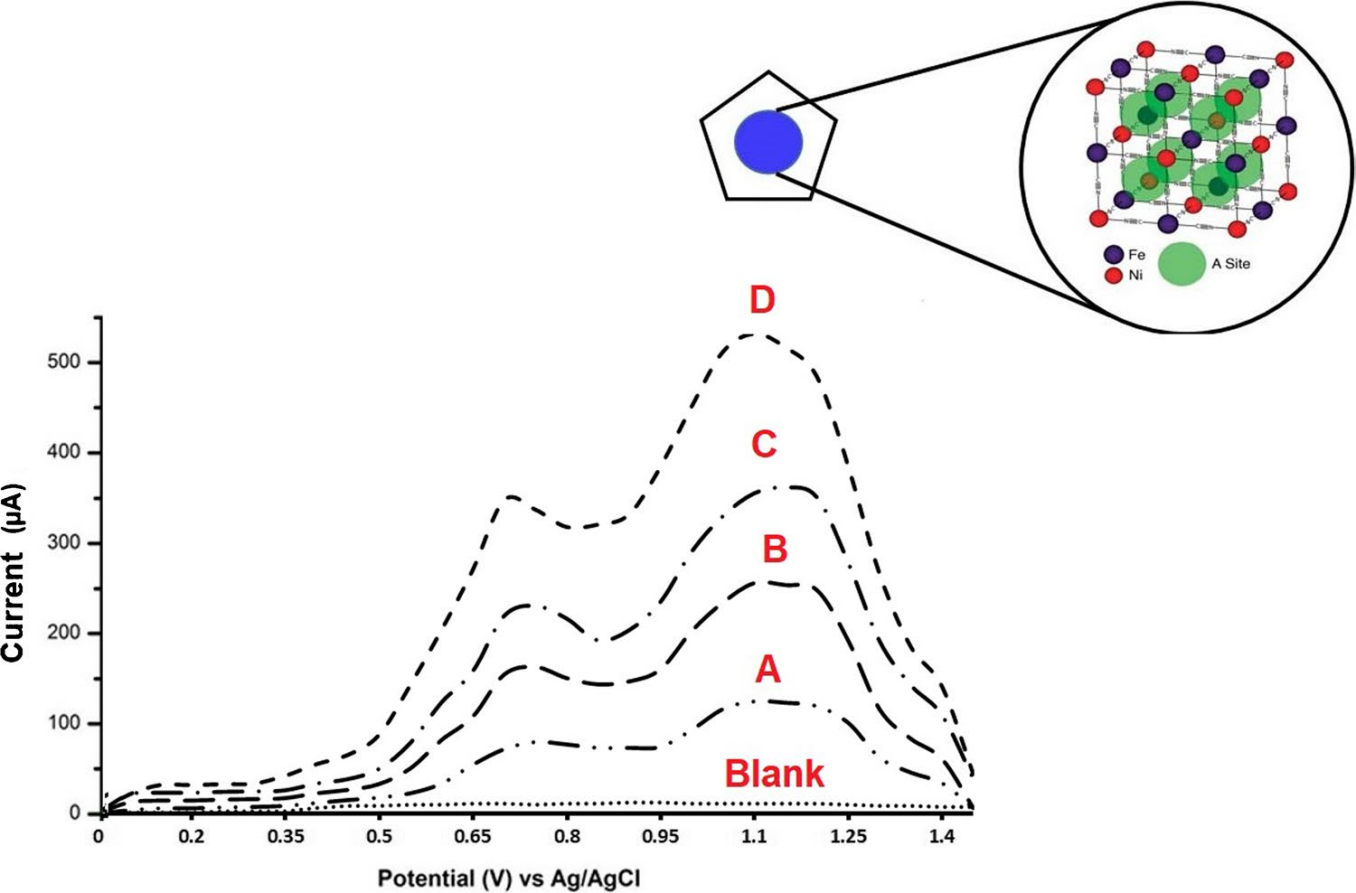
SWV curves of 10 × 10^−8^ mol L^−1^ of AMS recorded at (**A**) bare GCE, (**B**) bare PGE, (**C**) NiFe PBA Ns/PGE, and (**D**) p-DPG NCs@NiFe PBA Ns/PGE in 0.04 mol L^−1^ M BR buffer (pH 8.0) (represented as the dotted line) under the optimum conditions. Inset: schematic diagram of the NiFe PBA framework

The electrochemical performance of bare GCE, bare PGE, NiFe PBA Ns, and p-DPG NCs@NiFe PBA Ns was verified on the basis of the electro-oxidation behavior of AMS recorded by the SWV technique. As it is shown in Fig. 3, changes in the electrical conductivity of the polymer NCs/NiFe PBAs Ns are based on the charge transfer of NiFe PBA Ns incorporated with the conducting polymer matrix. It can be seen that AMS has two oxidation peaks at 0.76 and 1.1 V versus the Ag/AgCl electrode. Before surface modification (curves A, B), the bare electrodes showed a poor anodic wave, compared with the modified ones. The modification of electrodes using NiFe PBA Ns (curve C) resulted in an enhancement in the oxidation current intensity, which may be due to the increase in the active surface area, hence an increase in the charge transfer and conductivity of the deposited layers. As it is shown in curve D, the electrochemical behavior was greatly improved in more than threefold upon using p-DPG NCs@NiFe PBA Ns as surface modification, owing to the fast charge transport of carriers and ionic diffusion throughout the electrode leading to an improvement of the electrochemical behavior towards AMS on the modified electrode. Such a large shift of the current intensity of AMS oxidation peak indicates a great reduction in the activation energy of the drug electro-oxidation process, and thus the production of many new active sites in the modified sensor. This favorable response is achieved by the decoration of polymer nanocomposites incorporated with NiFe Ns that is supported with Prussian blue analogue.

### Investigation of the electrode reaction mechanism

**Fig. 4:**
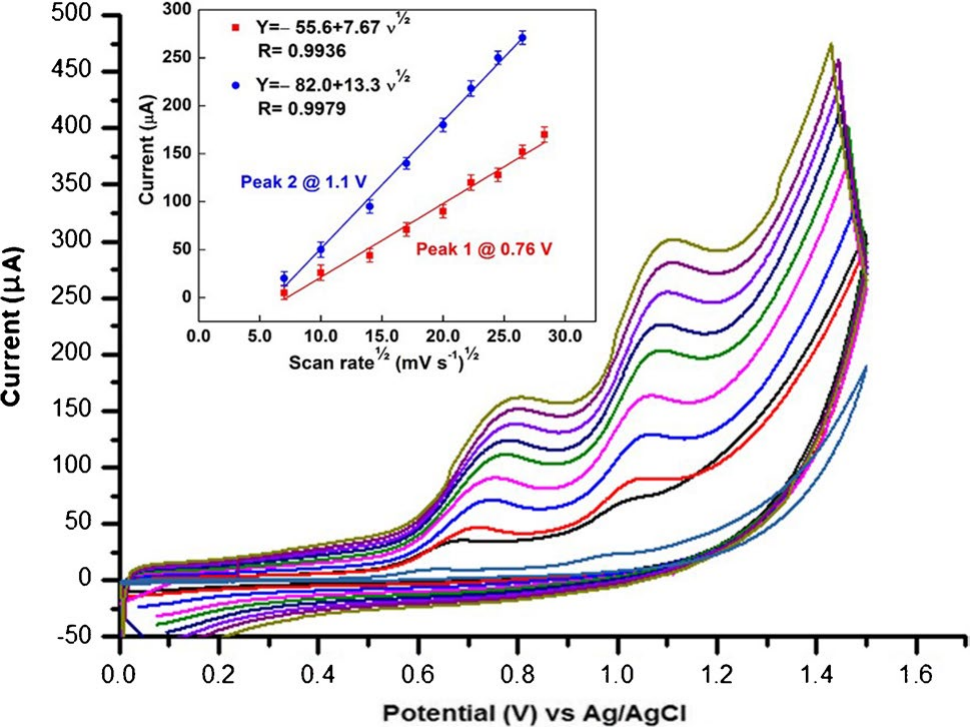
The effect of different scan rates (0.05–0.8 Vs^−1^) on cyclic voltammetric curves of 10 × 10^−8^ mol L^−1^ AMS. Inset: dependence of the oxidation peak current (*I*_p_/µA) of two oxidation peaks of AMS on the square root of the scan rate (mV s^−1^)^½^

There are two processes governing the electrode reactions, either adsorption or diffusion-controlled process, and the study of the impact of the scan rate (*ν*) on the recorded peak current (*I*_p_) can provide evidence to distinguish between these two processes. The effect of the scan rate on 10 × 10^−8^ mol L^−1^ of AMS in BR buffer at pH 8.0 at the p-DPG NCs@NiFe PBA Ns modified electrode was investigated by recording the related CVs at different scan rates from 0.05 to  0.8 V s^−1^. As shown from Fig. 4, the increase in the scan rate from 0.05 to 0.8 V s^−1^ is accompanied by a shift in the anodic oxidation peak potential of AMS to more positive values, with an increase in the current intensity indicating that the oxidation reaction of AMS is irreversible.

In order to study the mechanism of the electrochemical oxidation of AMS on the proposed electrode, the peak current (*I*_p_) was plotted against the square root of the potential scan rate (*ν*^½^). As shown in Fig. 4, a straight line was obtained, indicating that the oxidation reaction on the proposed sensor is controlled by diffusion process following these equations:

$$\begin{array}{l}\begin{array}{cc}{I}_{\mathrm{p}}\left(\mathrm{\mu A}\right)=-55.6+7.67{\upupsilon }^{1/2}& \left(\mathrm{R}=0.9936\right)\mathrm{ for\; Peak }\;1\end{array}\\ \begin{array}{cc}{I}_{\mathrm{p}}\left(\mathrm{\mu A}\right)=-82.0+13.3{\upupsilon }^{1/2}& \left(\mathrm{R}=0.9979\right)\mathrm{ for\; Peak }\;2\end{array}\end{array}$$

**Fig. 5:**
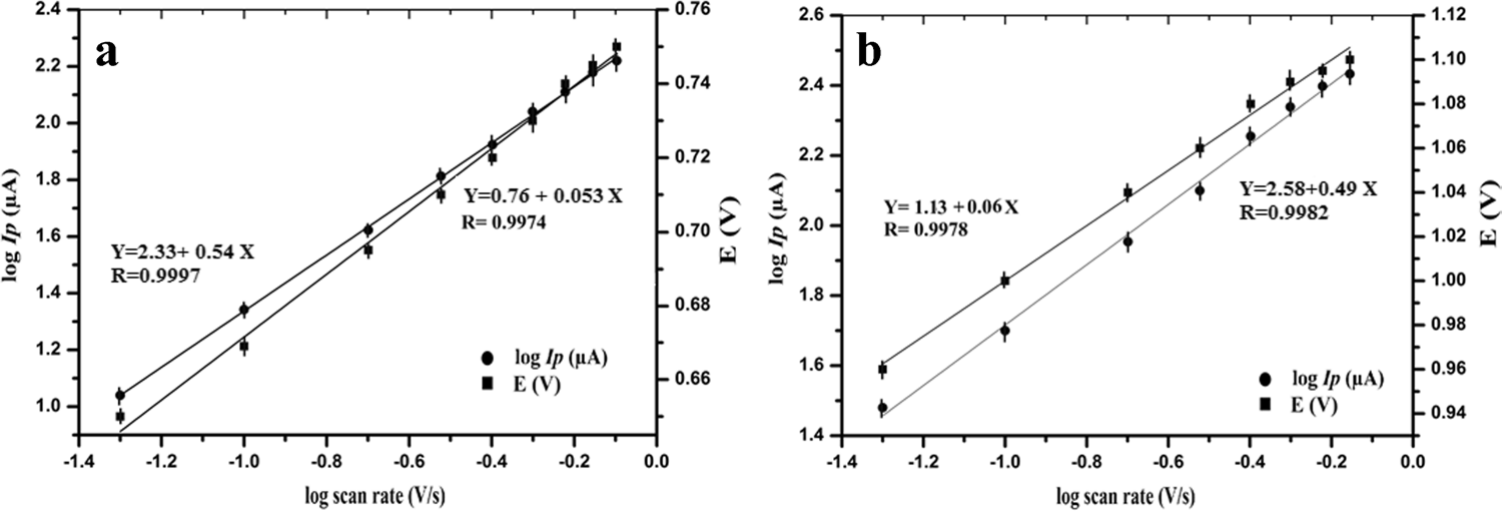
Dependence of the logarithm of peak current (log *I*_p_/μA) and the oxidation peak potential (*E*/*V*) on the logarithm of the scan rate (V s^−1^) for (**a**) first peak at 0.76 V and (**b**) second peak at 1.1 V

Figure 5a and b present the linear plot of log *I*_p_ versus the log of the scan rate (log *ʋ*) at the two oxidation peaks of AMS which can be described by the following regression equations:

$$\begin{array}{l}\begin{array}{cc}{\mathrm{log}I}_{\mathrm{p}}\left(\mathrm{\mu A}\right)=2.33+0.54\;\mathrm{\;log\;\upsilon }& \left(\mathrm{R}=0.9997\right) \mathrm{for \;peak \;}1\mathrm{ \;at\; }0.76\mathrm{ V}\end{array}\\ \begin{array}{cc}{\mathrm{log}I}_{\mathrm{p}}\left(\mathrm{\mu A}\right)=2.58+0.49\mathrm{\;log\;\upsilon }& \left(\mathrm{R}=0.9982\right) \mathrm{for\; peak\; }2\mathrm{ \;at }\;1.1\mathrm{ V}\end{array}\end{array}$$

The slope of the linear plot between log *I*_p_ and log *ʋ* was calculated to be 0.54 for peak 1 and 0.49 for peak 2, a result that is close to the theoretical value of 0.5 predicted by the Randles-Ševćik equation. These results emphasize a diffusion-controlled oxidation process of AMS which agreed with the previous studies [16, 17]. Additionally, the relationship between the peak potential *E* and the logarithm of the scan rate (log *ʋ*) was plotted producing a linear regression equation as follows:

$$\begin{array}{l}\begin{array}{cc}{E}_{\mathrm{p}}\left(\mathrm{V}\right)=0.76+0.053\;\mathrm{log\;\upsilon }& \left(\mathrm{R}=0.9974\right)\mathrm{ \;for\; peak\; }1\;\mathrm{ \;at }\;0.76\mathrm{ V}\end{array}\\ \begin{array}{cc}{E}_{\mathrm{p}}\left(V\right)=1.13+0.060\;\mathrm{log\;\upsilon }& \left(\mathrm{R}=0.9978\right)\mathrm{ \;for \;peak }\;2\mathrm{ \;at\; }1.1\mathrm{ V}\end{array}\end{array}$$

Since no cathodic peak was observed in the CV reverse scan, the involved irreversible reaction could be represented according to the Laviron theory for the totally irreversible electrode process. According to the Laviron equation [31], a relationship between the potential (*E*), scan rate, and number of electrons (*n*) transferred in the rate limiting step can be calculated from the following equation:

$$\mathrm{The\; slope\; of\; the \;plot }\;{E}_{\mathrm{p}}\left(\mathrm{V}\right)\mathrm{ \;and\;log}\;\upsilon =2.2303 RT/\alpha n\;F$$

*R* is the universal gas constant (8.314 J mol^−1^ K^−1^), taking *T* with 298 K, *α* is the transfer coefficient (= 0.5 in totally irreversible reactions), and *F* is the Faraday constant (96,480 C mol^−1^).

In order to calculate the number of involved electrons, after substituting the slope with 0.053 (the slope of the first anodic peak), the value of *αn* will be equal to 1.1 and the number of electrons is estimated to be 2.2 (≈ 2.0 electron). In the second peak, the slope is calculated to be 0.060 (the slope of the second anodic peak), the value of *αn* will be equal to 0.98, and the number of electrons is estimated to be 1.97 (≈ 2.0 electron). All the obtained results agree with the previously reported results concerning the oxidation of the pyrrolidine ring [16, 32].

### The suggested electro-oxidation mechanism of AMS

Some suggestions could be implied considering the oxidation behavior of AMS by studying the oxidation behavior of the potentially electro-active center anisidine ring (methoxyaniline). It was reported that the *m-*anisidine ring has one anodic oxidation peak, which is attributed to the oxidation of the primary amine group attached to the *m-*anisidine ring [16, 33]. Since the ethylsulfonyl group is inactive anodically, we can predict that the second anodic process of AMS at 1.1 V is attributed to the oxidation of the tertiary nitrogen atom in the pyrrolidine ring [34].

In order to have evidence on the previous suggestions, the FTIR spectra of intact and the oxidized form of AMS were recorded between 400 and 4000 cm^−1^. As this study is the first trial of elucidation of the AMS oxidation process using the FTIR technique, we took some reported researches on compounds having a structural similarity with AMS as a guide. Different characteristic FTIR peaks and chemical groups present in both intact and oxidized forms of AMS are shown in Fig. S2a. As can be seen, a peak at 3200 cm^−1^, assigned for N–H stretching of the amine group, in the spectrum of intact AMS which disappeared in the oxidized form. There is an appearance of a peak at 1550 cm^−1^ in the spectrum of oxidized AMS which could be assigned for the nitro group. Another difference between the two spectra is the disappearance of a peak at 1270 cm^−1^ which is assigned to the stretching of C–N and formation of a broad band at 3400 cm^−1^ which is due to stretching of the OH functional group. As shown in Fig. S2b, we can suggest that AMS undergoes an electro-oxidation process at the surface of the modified electrode on two positions, one at the amine group of the anisidine ring forming a nitro group. The second position is at the tertiary nitrogen group of the pyrrolidine ring forming the N–OH group [34].

### Optimization of experimental and electrochemical parameters

Optimization of several parameters, either experimental, such as type of buffer solution, and pH values, or instrumental affecting the oxidation peak current of AMS at the modified electrode, was investigated and presented in the supporting information section.

### Effect of supporting electrolyte type and pH

Different supporting electrolytes such as borate, phosphate, and Britton-Robinson (BR) buffer solutions at pH range over 5.5 to 9.0 were examined. From all these electrolytes, the BR buffer showed the highest oxidation peak current of AMS compared with other supporting electrolytes as represented in Fig. S3a (Supporting information). Further different concentrations of BR buffer were studied from 0.02 to 0.12 mol L^−1^. The best oxidation peak current was observed in the range from 0.03 to 0.08 mol L^−1^ BR buffer. Hence, 0.04 mol L^−1^ was selected as the optimum concentration for subsequent measurements.

Subsequentially, 0.04 mol L^−1^ BR buffer at pH 8.0 was selected as the optimum pH medium required for electro-oxidation of AMS. The linear relationship between potential and pH values of 0.04 mol L^−1^ BR buffer is shown in Fig. S3b (Supporting information). The oxidation peak potentials at 0.76 and 1.1 V were shifted towards negative potentials by increasing the pH value, indicating that the number of electrons transferred is similar to the number of protons involved in the electrode reaction as described previously.

### Square wave voltammetric (SWV) parameters

Different SWV parameters were studied, including pulse height within the range 3–30 mV, potential increment over the range from 1 to 30 mV, and frequency over the range from 25 to 250 Hz. It was found that 5 mV pulse height, 10 mV potential increment, and 225 Hz frequency were the optimum SWV parameters for the electro-oxidation of AMS on the modified electrode. Different accumulation potentials and accumulation times were investigated over the range of − 0.8 to + 1.5 V and from 10 to 90 s, respectively. It was observed that 0.0 V and 60 s as accumulation potential and time gave the highest peak current intensity and were selected as the optimum values.

### Response of p-DPG NCs@NiFe PBA Ns/PGE towards AMS determination

Different validation parameters, including linearity range, precision, accuracy, and sensitivity in the form of limit of detection and limit of quantitation, were investigated according to International Conference on Harmonization (ICH) guidelines [35].

**Fig. 6:**
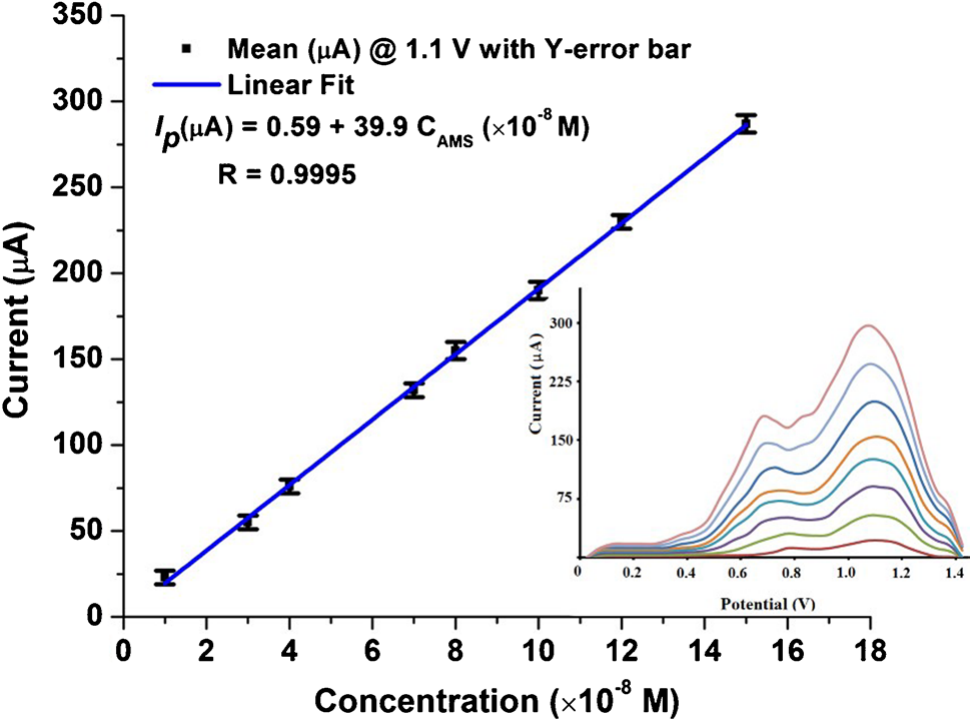
Calibration plots obtained for different concentrations of AMS (0.5 to 15 × 10^−8^ mol L^−1^) in 0.04 M BR buffer (pH 8.0) (SWV parameters, *E*_acc_ = 0.0 V, frequency = 225 Hz, pulse height = 5 mV, potential increment = 10 mV, *t*_acc_ = 60 s). Inset: SWVs of AMS monitored on p-DPG NCs@NiFe PBA Ns/PGE electrode

As shown in Fig. 6, the proposed SWV method was utilized for construction of the AMS calibration plot over a concentration range from 0.5 to 15 × 10^−8^ mol L^−1^ under the optimum experimental and instrumental conditions using 40 mV s^−1^ scan rate. The limit of detection (LOD = 3 *S*_*b*_/*m*) and the limit of quantitation (LOQ = 10 *S*_*b*_/*m*), where *S*_*b*_ is the standard deviation of the intercept and *m* is the slope of the calibration curve, were calculated to be 0.15 × 10^−8^ and 0.45 × 10^−8^ mol L^−1^, respectively. All the important quantitative statistical parameters of the proposed method are listed in Table S1. To ensure the validity of the proposed sensor, a comparison with other previous reported methods is presented in Table 1. The proposed method showed the least LOD among the reported methods, indicating higher sensitivity than the previously reported methods; some of these methods have several defects such as lack of selectivity, time consuming, or large consumption of organic solvents.

**Table 1:** Comparison of the proposed method with some previously reported studies for determination of AMS

Technique	Linearity range (μg mL^−1^)	LOD (μg mL^−1^)	Ref
RP-HPLC	2–10	0.0175	9
Spectrophotometry	1–24	0.36	10
Spectrophotometry	0.5 × 10^−3^–50	0.016	12
Fluorimetry	5–60 × 10^−3^	7 × 10^−4^	14
Voltammetric (stationary glassy carbon electrode)	1.48–22.2	0.81	16
Voltammetric (fsDNA biosensor)	1–10	0.46	17
The proposed sensor	**1.85 × 10**^**−3**^**–55.5 × 10**^**−3**^	**0.555 × 10**^**−3**^	**This work**

### Selectivity of p-DPG NCs@NiFe PBA Ns/PGE

**Fig. 7:**
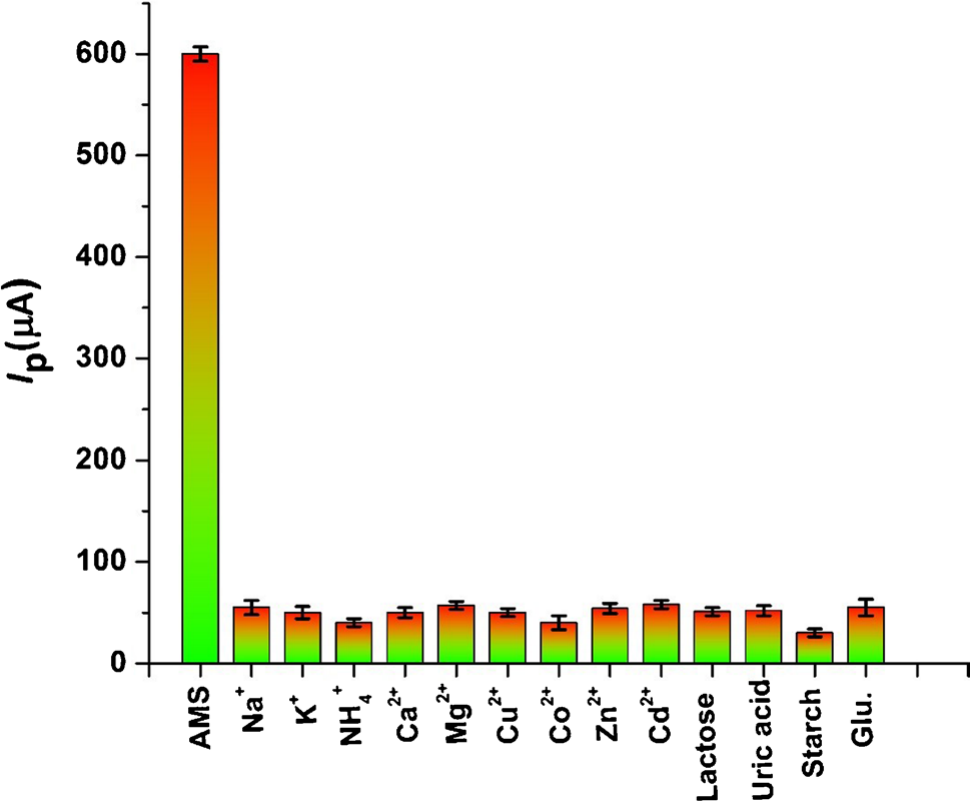
Histograms of SWVs of p-DPG NCs@NiFe PBA Ns/PGE in 0.04 mol L^−1^ M of BR buffer (pH = 8.0) containing 12 × 10^−8^ mol L^−1^ of AMS and different concentrations of biologically interfering compounds

The importance of the modified sensor for analysis is determined through its selectivity in the presence of different biologically interfering species. In order to evaluate the selectivity of p-DPG NCs@NiFe PBA Ns/PGE for the determination of AMS, it was treated with some potentially interfering substances. The tolerance limit for these interfering species was considered as the maximum concentration that gave a relative error less than ± 5.0% at a concentration of AMS under optimum conditions. As shown in Fig. 7, the results showed that 1000-fold excess of some biologically interfering ions such as Na^+^, K^+^, NH_4_^+^, Ca^2+^, and Mg^2+^ and some transition metals such as Cu^2+^, Co^2+^, Cd^2+^, and Zn^2+^, and 200-fold of some biologically interfering compounds such as lactose, magnesium stearate, starch, and sucrose, had no significant effect on the two oxidation peak currents of AMS. Also, 500-fold of various common interfering substances in biological samples such as glucose, glutathione (Glu.), uric acid, and ascorbic acid did not affect the anodic peaks of AMS (signal change less than 4%). The results obtained showed recoveries over the range 96.3–102.1%, indicating that AMS can be determined in pharmaceuticals and biological samples with high selectivity without any interference from most common interfering substances using the modified electrode.

### Stability and reproducibility of p-DPG NCs@NiFe PBA Ns/PGE

Stability of p-DPG NCs@NiFe PBA Ns/PGE was investigated using the CV technique by applying 50 cycles at 150 mV s^−1^ scan rate on the 5 mmol L^−1^ Fe^2+^/Fe^3+^ system model. Figure S4a inferred the stability of p-DPG NCs@NiFe PBA Ns/PGE as indicated from the unchanged *I*_*p*_ values during the continuous scans. Additionally, the long-term stability of p-DPG NCs@NiFe PBA Ns/PGE was investigated by storing the electrode at 4 °C, and it was applied for AMS determination every 5 days up to 20 days. The obtained results presented in Fig. S4b show that the current response decreased only by 5% of its initial activity after 20 days of application. So, it can be inferred that the modified electrode is suitable for the long-time analysis. To study the reproducibility of the modified electrode, five independent sensors were fabricated and used to determine AMS. The RSD % value did not exceed 3.0% which proves the good reproducibility of p-DPG NCs@NiFe PBA Ns/PGE (Fig. S4c).

### Determination of AMS in the presence of COVID-19 co-administered drugs

**Fig. 8:**
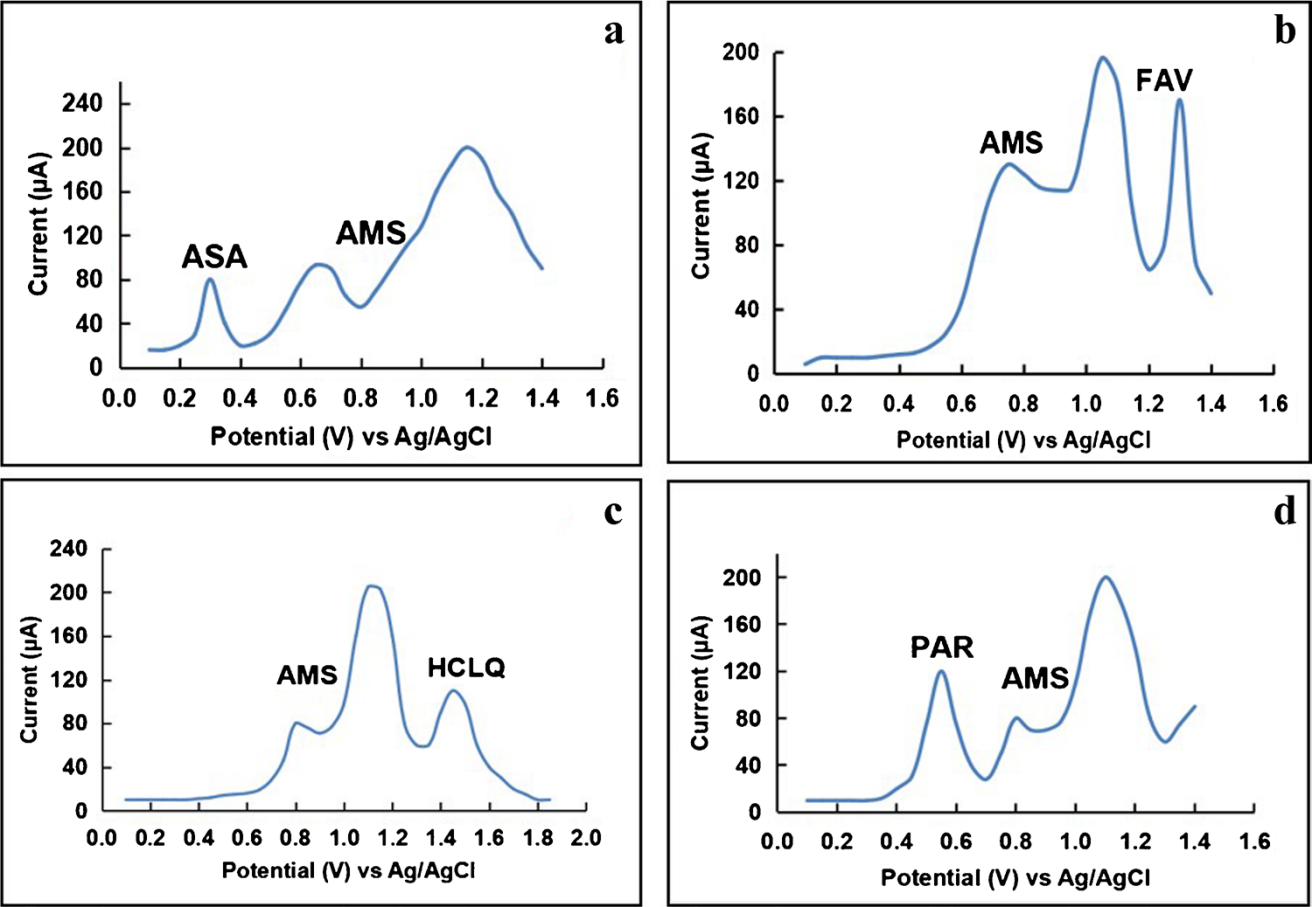
Square wave voltammograms using p-DPG NCs@NiFe PBA Ns/PGE sensing platform in BR buffer (pH 8.0) containing 5 × 10^−8^ mol L^−1^ of AMS, (**a**) 1 × 10^−4^ mol L^−1^ of ASA, (**b**) 1 × 10^−4^ mol L^−1^ of FAV, (**c**) 1 × 10^−5^ mol L^−1^ of HCLQ, and (**d**) 1 × 10^−5^ mol L^−1^ of PAR, for each at scan rate 40 mV s^−1^, pulse height = 5 mV, potential increment = 10 mV, and *t*_acc_ = 60 s

One of the important targets of the proposed platform is to investigate its capability of accurate tracing of the related analysis in the presence of other co-exciting significant analytes. The modified platform was successfully utilized for tracing AMS in the presence of four COVID-19 drugs of different pharmacological actions. It was reported that AMS is primarily eliminated via the renal route and has a limited degree of metabolism, as 80% of the eliminated drug is in the parent form and its metabolites are not detectable in plasma [36]. Owing to the limited interference of AMS metabolites and according to the Egyptian national guidelines for COVID-19, hydroxychloroquine (HCLQ), ascorbic acid (ASA), favipiravir (FAV), and paracetamol (PAR) have been selected to carry out the selectivity study of the modified platform. ASA, an antioxidant possessing anti-inflammatory properties, affects cellular immunity and vascular integrity [37]. Figure 8a shows well-resolved anodic peaks at + 0.30 V, + 0.77 V, and + 1.1 V (*vs*, Ag/AgCl) corresponding to the oxidation of ASA and AMS, respectively [38]. FAV shows an activity in preventing viral replication by selectively inhibiting the RNA polymerase of RNA viruses [39], giving a well-resolved oxidation peak at + 1.3 V (Fig. 8b) that is well matched with the previously reported work [40]. HCLQ is being taken for post-exposure prophylaxis to prevent progression to symptomatic disease after COVID-19 exposure, and gave a high-resolution anodic peak along with good sharpness (Fig. 8c) [41]. PAR is one of the most common painkillers and non-steroidal anti-inflammatory actions that is used globally due its safety, showing a well-resolved oxidation peak at + 0.53 V, while the anodic peak of AMS was achieved at + 0.77 V (Fig. 8d) [42]. Referring to Fig. 8, it is clear that the SWVs of AMS with one of the COVID-19 drugs (PAR, ASA, FAV, and HCLQ) were monitored under the optimized experimental conditions showing an excellent anodic peak separation that allows the precise and selective determination of AMS, indicating the high accuracy and precision of the modified electrode for simultaneous determination of AMS with other COVID-19 drugs.

### Application of p-DPG NCs@NiFe PBA Ns/PGE

The reliability of the modified sensor for the determination of AMS was investigated in different biological samples. The results listed in Table 2 confirm the efficiency of p-DPG NCs@NiFe PBA Ns/PGE for determination of AMS in human plasma and urine samples. The values of the recovery percentages for quantitation of AMS indicate the feasibility and the accuracy of the developed surface modification for AMS determination in biological samples without any interference from co-existing substances in the plasma sample after a simple extraction method [43]. The results obtained confirm the suitability and sensitivity and gave an evidence that the proposed platform has an extreme potential for the analysis of low and high concentrations of AMS in different real samples. The represented study was performed in accordance with the Declaration of Helsinki [44].

**Table 2:** Determination of AMS in spiked human plasma and urine by the proposed SWV method

Sample	Added concentration (× 10^−8^ mol L.^−1^)	Found concentration (× 10^−8^ mol L.^−1^)	% Recovery ± SD^a^	RSD %		
Plasma	2.0	1.93	96.50 ± 1.25	2.23		
	4.0	3.92	98.40 ± 2.14	1.74		
	8.0	7.85	98.13 ± 1.36	1.56		
	12.0	11.66	97.16 ± 2.45	1.82		
	14.0	13.65	97.50 ± 2.51	2.34		
Urine	2.0	2.01	100.5 ± 0.56	2.13		
	4.0	3.89	97.25 ± 1.25	1.89		
	8.0	7.99	99.86 ± 1.89	1.74		
	12.0	12.14	101.16 ± 2.01	1.58		
	14.0	13.75	98.21 ± 1.37	1.69		
	Proposed method	Reported method^b^	% Recovery ± SD^a^	RSD %	*t* test^bc^	*F* test^bc^
Solian R tablets	8.0	7.95	99.37 ± 0.85	1.15	0.685	1.326
	10.0	10.01	100.10 ± 0.99	1.66		
	12.0	11.76	98.00 ± 1.23	2.06		

^a^Average of 6 determinations

^b^Ref. 16

^c^Theoretical values at 95% confidence limit; *t* = 2.228, *F* = 5.053

## Conclusion

In order to identify and avoid the danger of serious drug-drug interactions that may occur in COVID-19 patients taking antipsychotic drugs, a simple, efficient, and convenient electrochemical sensor has been described for careful monitoring and trace determination of AMS levels in human plasma. The voltammetric sensor is based on a pencil graphite electrode modified with *in situ* electro-polymerized glyoxal polymer nanocomposites incorporated in bimetallic (Ni–Fe) Prussian blue analogue nanopolygons framework. The synergistic effect of the conductivity of the electro-polymerized polymer nanocomposites with the open framework of PBAs has been utilized to enhance the electrochemical signal of the modified electrode *via* increasing the active surface area and increasing the active sites leading to facilitate the charge transfer of the interested analyte. Additionally, this novel sensor exhibits not only good sensitivity, and selectivity for AMS determination in the presence of different interfering species, but also excellent reproducibility and stability. The modified sensor displays a linear response towards AMS oxidation over 0.5 to 15 × 10^−8^ mol L^−1^ concentration range, with 1.5 nmol L^−1^ detection limit. Stepwise assembly of the modified electrode was further characterized using SEM, TEM images, PXRD, EIS, CV, and SWV techniques. Additionally, p-DPG NCs@NiFe PBA Ns/PEG platform was able to selectively detect AMS in the presence of some co-administered COVID-19 drugs. Finally, this sensor has been applied successfully to detect AMS in spiked human plasma and urine samples, indicating the potential of the fabricated p-DPG NCs@NiFe PBA Ns/PEG sensor for practical clinical applications. This sensing system opens up a new avenue for utilizing the modified platform to further studies of the drug-drug interactions and the pharmacokinetic of AMS with more prescribed COVID-19 medications.

### Supplementary Information

Below is the link to the electronic supplementary material.

Supplementary file1 (DOCX 735 KB)
