# Correction to: NiFe‑based Prussian blue analogue nanopolygons hybridized with functionalized glyoxal polymer as a voltammetric platform for the determination of amisulpride in biological samples

**DOI:** 10.1007/s00216-024-05144-9

**Published:** 2024-01-17

**Authors:** Marwa R. El‑Zahry, Marwa F. B. Ali

**Affiliations:** 1 Pharmaceutical Analytical Chemistry Department, Faculty of Pharmacy, Assiut University, Assiut, 71526 Egypt; 2 Pharmaceutical Chemistry Department, Faculty of Pharmacy, Badr University in Assiut, Assiut, 2014101 Egypt

**Correction to** **:** **Analytical and Bioanalytical Chemistry (2023) 415:1559–1570**

10.1007/s00216-023-04559-0

The original article contained a mistake.

1. The section heading "Real-sample analysis" should be changed to "Spiked-sample analysis".
2. The sentence and phrase below should be deleted in the text:
  - "The represented study was performed in accordance with the Declaration of Helsinki and approved by the Egyptian Network of Research Ethics Committees (ENREC) (No. NCT04363229)."
  - To delete "in real samples" in the section heading: "Application of p-DPG NCs@NiFe PBA Ns/PGE in real samples"
  - "and approved by the Egyptian Network of Research Ethics Committees (ENREC) (No. NCT04363229)."
3. The sentence "The represented study was performed in accordance with the Declaration of Helsinki [44]" should be placed in the section "Application of p DPG NCs@NiFe PBA Ns/PGE".
4. Under the section heading "Real-sample analysis": The sentence “Human urine and plasma samples were provided by Assiut University Hospitals (Assiut, Egypt)” should be changed to “Human urine and plasma samples from the blood bank have been granted by Assiut University Hospitals (Assiut, Egypt). The pooled plasma samples have been collected from healthy volunteers and stored under -20° C until use”.

