# Supplementary material for: NiFe-based Prussian blue analogue nanopolygons hybridized with functionalized glyoxal polymer as a voltammetric platform for the determination of amisulpride in biological samples
Source: Anal Bioanal Chem. 2023 Feb 21;415(8):1559–70. doi: 10.1007/s00216-023-04559-0 (PMC9942618; doi:10.1007/s00216-023-04559-0)
Supplement: Supplementary file 1 — Supplementary file1 (DOCX 735 KB) [file 216_2023_4559_MOESM1_ESM.docx]

**Electronic Supplementary Data**

NiFe-based Prussian blue Analogue Nanopolygons Hybridized with Functionalized Glyoxal Polymer Voltammetric Platform for Determination of Amisulpride in Biological Samples

**Marwa R. El-Zahry^1, 2*^ and Marwa F. B. Ali^1^**

*^1^ Pharmaceutical Analytical Chemistry Department, Faculty of Pharmacy, Assiut University, Assiut 71526, Egypt*

*^2^ Pharmaceutical Chemistry Department, Faculty of Pharmacy, Badr University, Assiut 2014101, Egypt*

*Corresponding author: Department of Pharmaceutical Analytical Chemistry, Faculty of Pharmacy, Assiut University, 71526 Assiut, Egypt

Email: [marwazahry@aun.edu.eg](mailto:marwaali1@aun.edu.eg)

Fax number: 0020-88-2080774


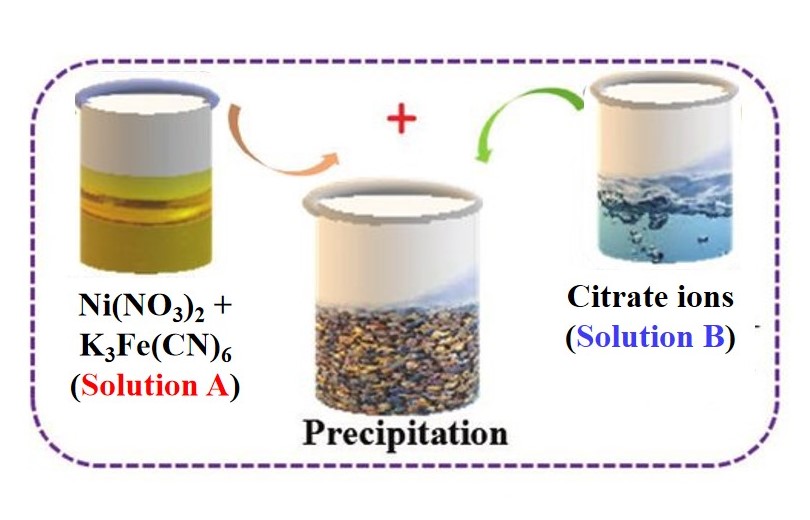


**Scheme S1** The fabrication process of PBAs frameworks precipitation


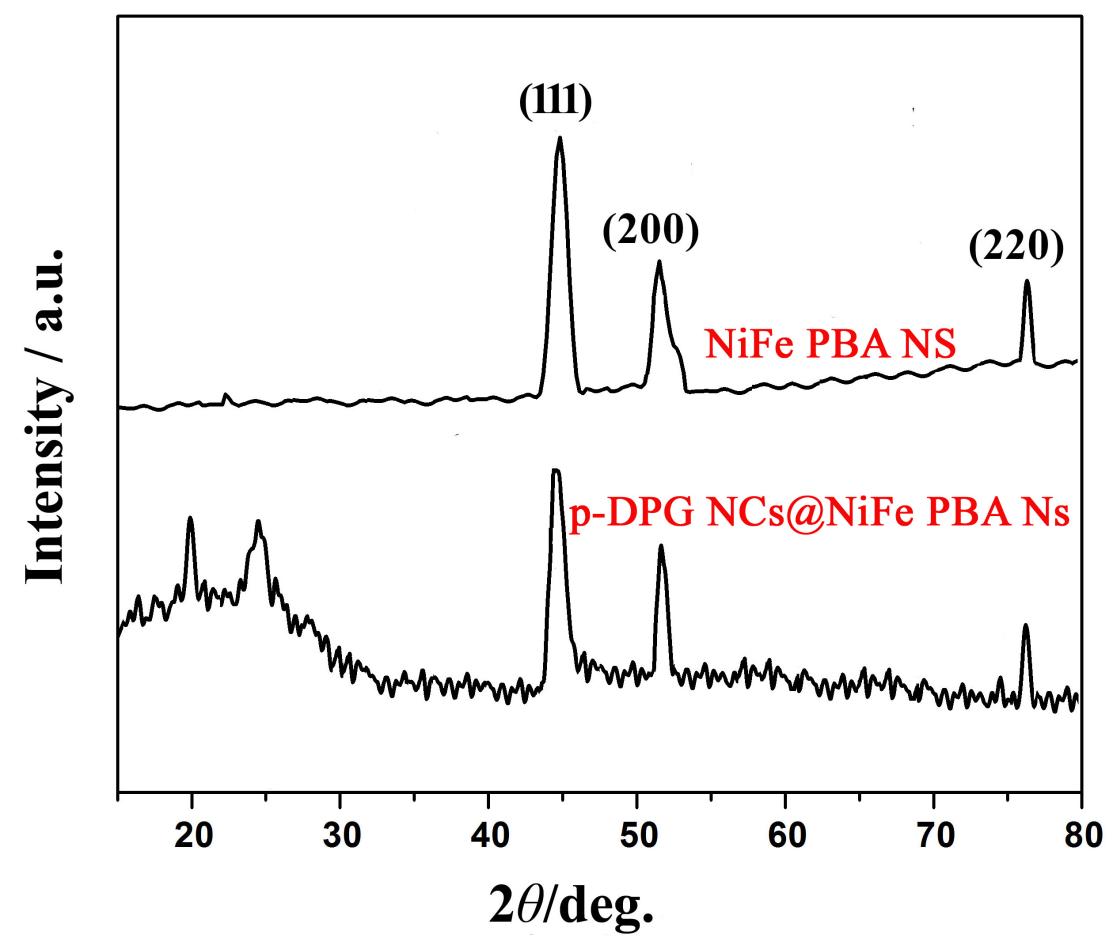


**Fig S1** PXRD patterns of NiFe PBA Ns and p-DPG NCs@NiFe PBA Ns


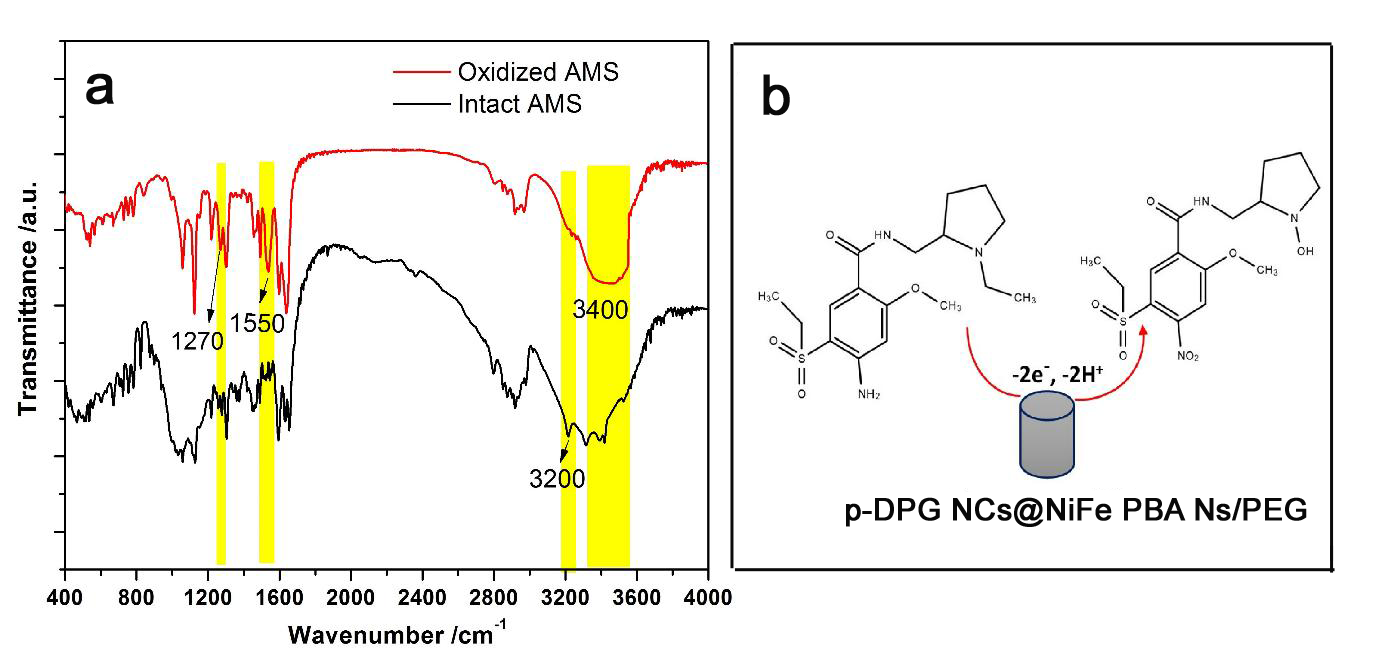


**Fig S2** (a) FTIR spectra of the oxidized and intact forms of AMS, (b) Suggested oxidation mechanism of AMS on the modified electrode

**
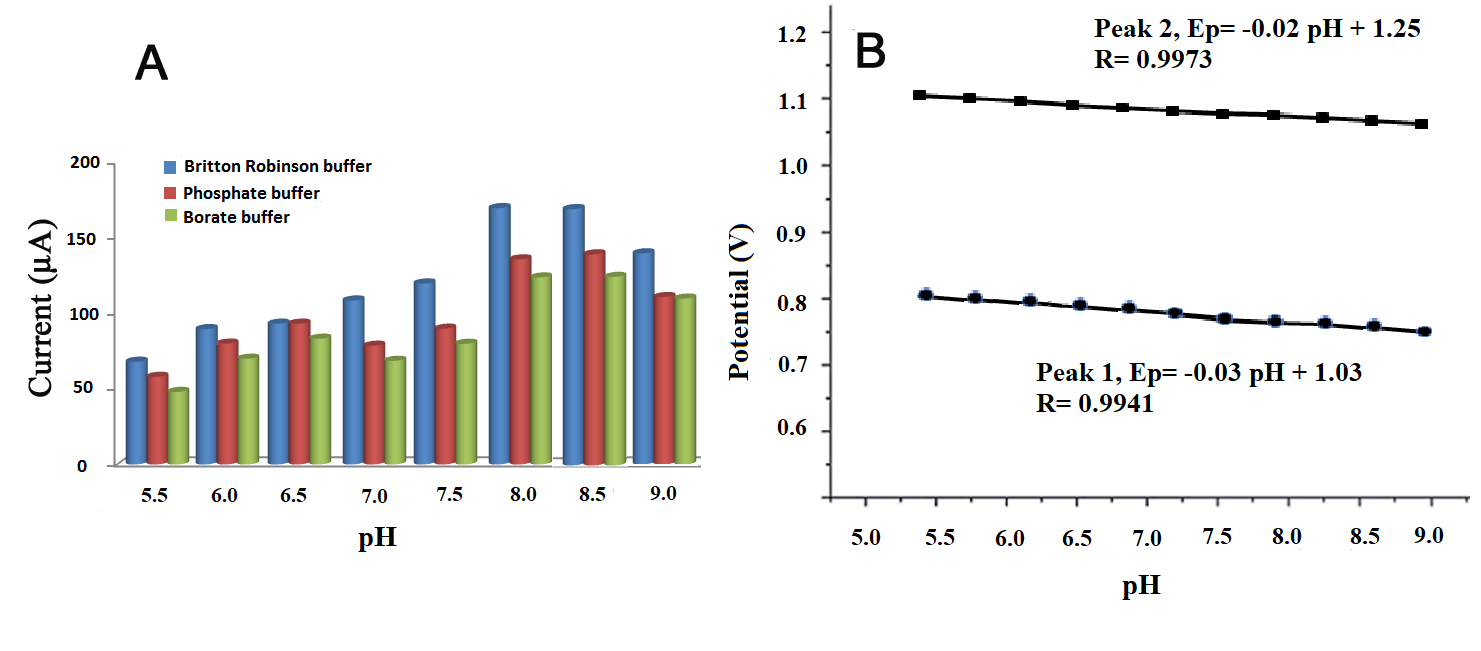
**

**b**

**a**

**Fig S3** (a) Effect of different types of supporting electrolytes and different pH values on current intensity of 4.0 ×10^−8^ mol L^−1^AMS under the optimum conditions, (b) Linear plot between potential (V) and pH values of 0.04 mol L^−1^ BR buffer at the two oxidation peaks of AMS.


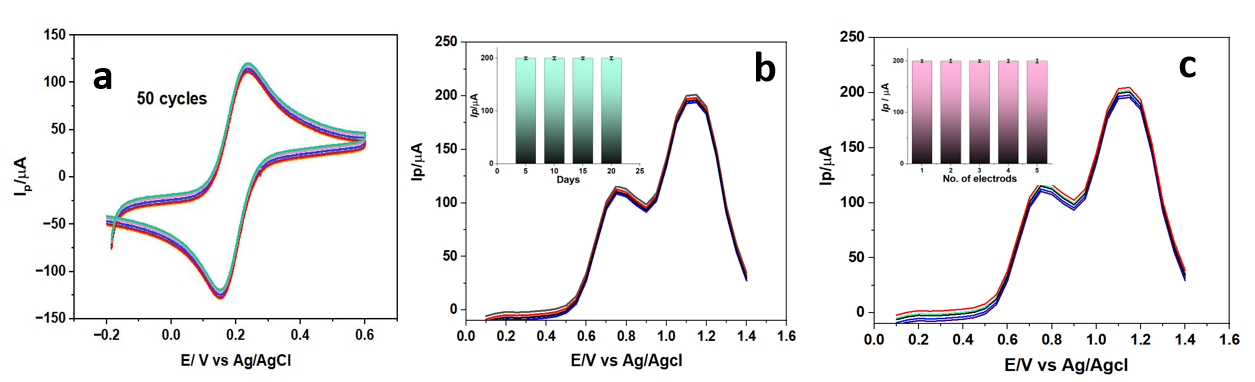


**Fig S4** (a) CV scans of p-DPG NCs@NiFePBA Ns/PGE for 50 successive cycles. (b) SWV scans of four prepared p-DPG NCs@NiFePBA Ns/PGE in BR (pH 8.0) containing 5.0 ×10^−8^ mol L^−1^. Inset of (b) is histogram. (c) SWV scans of the prepared p-DPG NCs@NiFe PBA Ns/PGE in 5.0 ×10^−8^ mol L^−1^AMS. Inset of (c) is histogram.

| Parameters | AMS |
| --- | --- |
| Linearity range (× 10^−8^ mol L^−1^) | 0.5 - 15 |
| Intercept (μA) ± SD ^a^ | 0.59 ± 0.38 |
| Slope (μA.mol^−1^L) ± SD ^b^ | 39.9 ± 0.05 |
| Correlation coefficient (R) | 0.9995 |
| LOD ^c^ (× 10^−8^ mol L^−1^) | 0.15 |
| LOQ ^d^ (× 10^−8^ mol L^−1^) | 0.45 |
| Intra-day precision ^e^ (% RSD) n=5^f^ | ≤ 2.65 |
| Inter-day precision ^e^ (% RSD) n=5^f^ | ≤ 2.44 |

**Table S1** Quantitative statistical parameters of AMS using the proposed SWV method

^a^ Standard deviation of intercept, ^b^ Standard deviation of slope, ^c^ limit of detection, ^d^ limit of quantitation, ^e^ Relative standard deviation, ^f^ Mean values of five measurements at three different concentration levels
